# KKI-NECT: Kennedy krieger institute’s network for early childhood tele-education (US)

**DOI:** 10.1192/j.eurpsy.2021.165

**Published:** 2021-08-13

**Authors:** M. Leppert, J. Harrison

**Affiliations:** 1 Kennedy Krieger Institute, Center For Development And Learning, Johns Hopkins School of Medicine, Baltimore, United States of America; 2 Kennedy Krieger Institute, Department Of Psychiatry, Johns Hopkins School of Medicine, Baltimore, United States of America

**Keywords:** mental health disparities, child and adolescent psychiatry, mental health care

## Abstract

**Introduction:**

The Kennedy Krieger Institute Network for Early Childhood Tele-Education (KKI-NECT) is a federally funded ECHO project. Its hub consists of a child psychiatrist, developmental pediatricians and a behavioral psychologist. Its community partners are primary care providers(PCPs) in underserved areas. Its goal is to create local experts in early childhood behavioral, emotional and developmental disorders.

**Objectives:**

After attendance at this session, the learner will be able to: 1. report the rates of co-occurring developmental, behavioral and emotional disorders presented by primary care participants 2. explain the efficacy of case based learning and a structured curriculum as a mechanism for expanding the workforce. The goal of this presentation is to build awareness of and interest in ECHOs specifically targeted to child behavioral, emotional and developmental issues.

**Methods:**

Dr. Leppert will discuss KKI-NECT, particularly the process of procuring funding, setting up an ECHO, and getting institutional “buy-in". She will describe the use of case based learning and a structured curriculum in a longitudinal CME program, report the comorbidities in cases that participants present for discussion, and demonstrate the impact on participants’ practice.

**Results:**

Data from four cohorts demonstrate that PCPs showed increased comfort levels, improved knowledge of behavioral, emotional and developmental disorders. PCPs expanded the scope issues they could address in their practice as a result of participation in KKI-NECT.

**Conclusion:**

KKI-NECT is a viable response to the workforce shortage of child psychiatrists by confidently increasing the role of the PCP in treating childhood developmental and mental health disorders.

**Disclosure:**

No significant relationships.

